# Outcomes and safety of colonoscopy in elderly patients aged 80 years and older: a retrospective cohort study

**DOI:** 10.7717/peerj.20404

**Published:** 2025-11-19

**Authors:** Yiming Ding, Xiangchun Lin

**Affiliations:** Department of Gastroenterology, Peking University International Hospital, Beijing, China

**Keywords:** Elderly, Individuals aged 80 years and older, Colorectal cancer, Colonoscopy

## Abstract

**Background:**

The incidence of colorectal tumors increases with age. Colonoscopy is crucial for early detection, yet performing it on patients aged 80 and older poses challenges due to their complex health conditions.

**Methods:**

This retrospective study included 447 elderly patients aged 80 years and older who underwent colorectal examinations at Peking University International Hospital from November 2015 to June 2024. Data on demographics, comorbidities, smoking and drinking history, and colonoscopy indications were collected. Risk factors for non-colorectal malignant tumors, colorectal malignant tumors, and colorectal advanced-stage tumors were compared. All patients received standard bowel preparation, and procedures were conducted by experienced endoscopists.

**Results:**

A total of 364 patients (81.4%) completed the procedure successfully. The main reasons for failure were inadequate intestinal preparation and intestinal stenosis. A total of 37 (8.3%) had no significant abnormalities, 315 (70.5%) were diagnosed with colorectal polyps, and 110 (24.6%) had malignant tumors. The complication rate was 0.67%. Univariate analysis showed that patients with colorectal malignant tumors were older (*P* = 0.000) and had a higher prevalence of alcohol consumption history (*P* = 0.014). For advanced tumors, patients were also older (*P* = 0.000).

**Discussion:**

Age is a significant risk factor for colorectal malignancies and advanced tumors. Although colonoscopy in elderly patients has acceptable safety, they face challenges in bowel preparation and examination techniques. The incidence of polyps and malignant tumors in this population is high. However, the study has limitations, such as its retrospective nature and reliance on incomplete electronic medical records.

**Conclusion:**

Colonoscopy is effective and safe for elderly patients aged 80 and older. It is essential for detecting colorectal malignancies and polyps. Age-related risk factors highlight the importance of this procedure in this high-risk group.

## Introduction

With the advancement of medical standards and economic strength, the issue of an aging population has become increasingly severe. As life expectancy rises, clinicians often encounter challenges when treating elderly patients. A growing number of individuals aged 80 and older are seeking medical attention. As age increases, there is a corresponding rise in basic complications and a decline in the functionality of various organs. Additionally, the incidence of tumors escalates with advancing age (Lin et al., 2006; Brenner et al., 2024); colorectal tumors, in particular, rank as the third most common malignant neoplasms in the world.

Colonoscopy is considered the gold standard for diagnosing colorectal cancer (Kahi et al., 2014). However, elderly patients represent a unique subset of individuals undergoing this procedure, which may also elevate the associated risks (Shafrir et al., 2019). This study aims to evaluate the impact of colonoscopy and analyze its results in adults aged 80 years and older.

## Materials and Methods

### Population included

**Figure 1:**
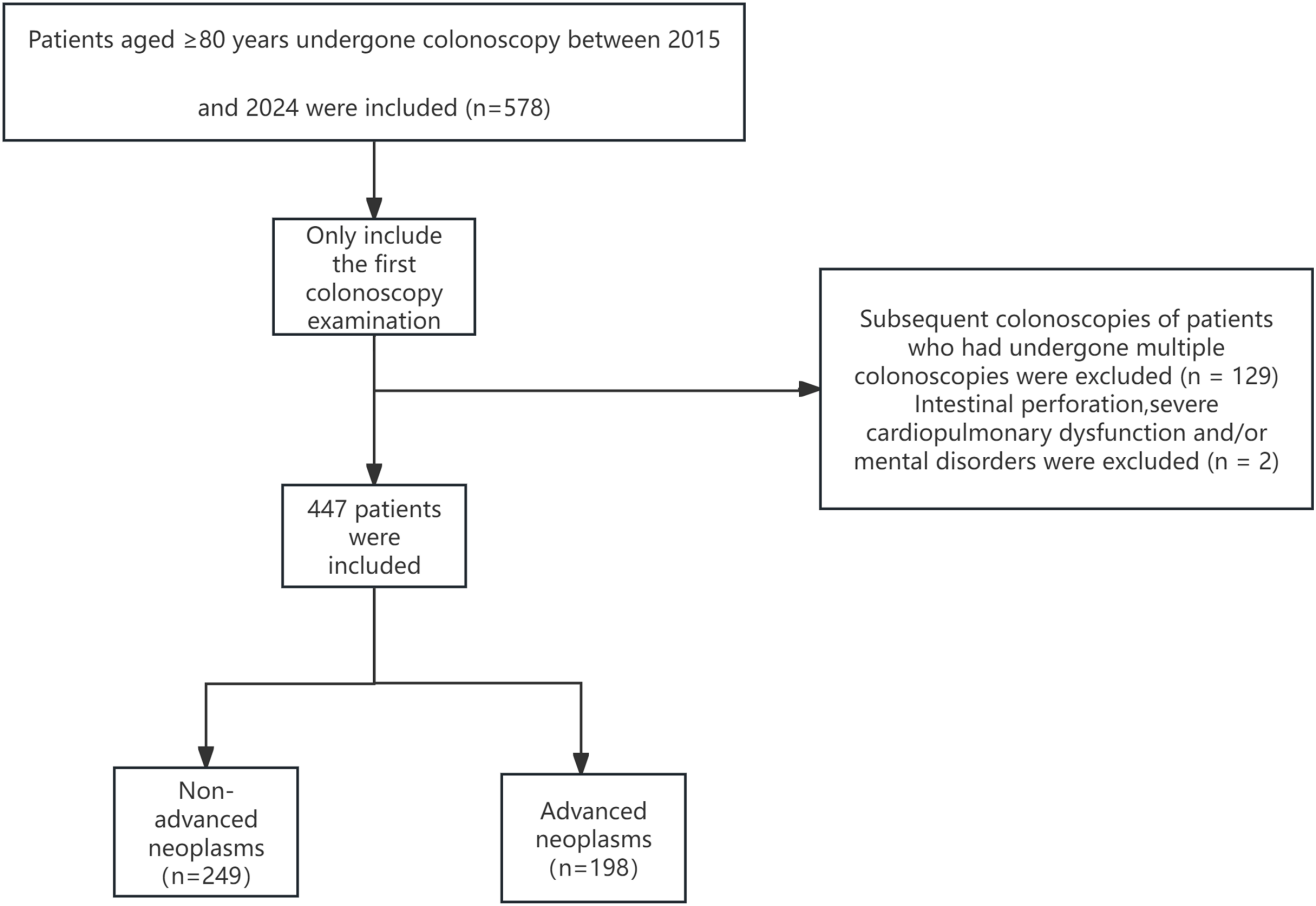
Flowchart of patient enrollment.

This study involved patients who underwent colonoscopy at the Endoscopy Center of Peking University International Hospital from November 2015 to June 2024. Patients aged 80 years and older were included. All participants received standard bowel preparation, usually with four boxes of polyethylene glycol electrolyte powder. In cases where oral bowel cleansing agents were not feasible, mannitol or saline enemas were used. All procedures were performed by experienced endoscopists. Only the initial colonoscopy data of each patient aged ≥80 years were included, and subsequent examinations were excluded. We obtained the above-mentioned data on October 28th, 2024 (Fig. 1).

A complete colonoscopy refers to a procedure in which the colonoscope is inserted through the anus, advanced through the rectum and sigmoid colon, and extended to the terminal ileum, enabling direct and clear visualization of the entire colonic mucosa. In this research, the painless endoscopic examination utilized intravenous anesthesia techniques. The primary anesthetic drugs consisted of propofol, fentanyl, and etomidate. Specifically, the average dosage of propofol was 1–2 mg/kg, with the total dosage controlled within 100–200 mg; the dosage range of fentanyl was 0.7–1.5 μg/kg, and the total dosage ranged from 50 to 150 μg; the dosage of etomidate was 0.2–0.3 mg/kg, and the total dosage was approximately 15–20 mg. The elderly patients involved in this study were examined by 10 endoscopy physicians. Among them, six had over 10 years of experience in colonoscopy procedures and an average of more than 1,000 colonoscopy examinations per year; the remaining four had 4–7 years of colonoscopy experience and an average of 500–800 colonoscopy examinations per year.

All endoscopists involved in this study were highly experienced professionals, each having completed more than 2,000 colonoscopy procedures, with an annual procedural volume exceeding 500. The Boston Bowel Preparation Scale was employed to assess the quality of bowel preparation among patients. A score of 6 or higher was classified as good or excellent, whereas a score below 6 was considered inadequate.

Adverse events encompass those arising from the bowel preparation process or the surgical procedure itself within 30 days following colonoscopy. These events were documented through a thorough review of medical records. Adverse events were categorized into gastrointestinal incidents (such as bleeding or perforation), major cardiopulmonary complications (including myocardial infarction, respiratory failure, or symptomatic arrhythmias), and minor cardiopulmonary issues (such as asymptomatic transient decreases in oxygen saturation to <90%, transient hypotension, or asymptomatic arrhythmias). Any emergency visit occurring within 30 days was classified as an adverse event. Serious adverse events were defined as any major cardiopulmonary complication, bleeding or perforation subsequent to polypectomy, or any complication necessitating unplanned hospitalization, blood transfusion, or termination of surgery.

### Exclusion criteria

Patients exhibiting clear intestinal perforation, significant cardiopulmonary dysfunction, or mental disorders that impaired cooperation during the colonoscopy procedure were excluded from this study.

### Research indicators

The study examined various research indicators, including age, gender, smoking and drinking history, family history of colorectal cancer, hyperlipidemia (cholesterol, triglycerides, low-density lipoprotein), glycosylated hemoglobin levels, hypertension, coronary heart disease, type 2 diabetes mellitus, height and weight measurements, the purpose of colonoscopy procedures, complications associated with colonoscopy (such as bleeding and perforation), and mortality within 30 days post-procedure.

### Clinical data collection

Clinical data were carefully collected from the electronic medical records of selected patients. This included clinical notes, emergency room documentation, examination reports, admission records, anesthesia logs, surgical records, and discharge summaries. Indications for colonoscopy procedures and bowel preparation medications used were documented. Additional information collected covered colonoscopy completion rates, bowel preparation quality assessments, procedure findings, immediate adverse events, and technical difficulties. Colorectal malignancy was defined as a lesion containing a carcinomatous component. Advanced neoplasms were characterized by tubular adenomas (TA) measuring ≥10 mm or any adenoma with villous features or high—grade dysplasia; sessile serrated polyps (SSP) measuring ≥10 mm or any SSP with dysplasia; conventional serrated adenomas; or invasive adenocarcinoma. The shape, size, number, and location of all detected polyps were systematically recorded.

### Statistical methods

For normally distributed variables, patient data and clinical parameters are presented as the mean plus standard deviation. In contrast, for non-normally distributed variables, these data are reported as the median. Categorical variables are expressed as absolute numbers with corresponding population proportions (percentages) indicated in brackets. To assess differences in the distribution of categorical data, we employed either the $\chi^2$ test or Fisher’s exact test, depending on the context. Continuous variables were analyzed using a t-test for normally distributed data and Mann-Whitney U test for non-normally distributed data; a *P*-value of less than 0.05 was considered indicative of statistical significance. All calculations were conducted using SPSS version 26.0 software (IBM Corp., Armonk, NY, USA). The statistical analysis of the research is scheduled to be completed in November 2024.

### Ethical review board approval

The study protocol adhered to the ethical guidelines of the 1975 Declaration of Helsinki. It was approved by the Biomedical Ethics Committee of Peking University International Hospital on October 15, 2024, with the approval number 2024-KY-0093-01. Due to the retrospective nature of the study and the lack of obvious potential harm to enrolled patients, the institutional review board waived the requirement for informed consent. The data used were retrieved from an existing database, and patient identities were anonymized and de-identified.

## Results

From November 2015 to June 2024, a total of 447 patients aged 80 years and older who met the inclusion and exclusion criteria underwent colonoscopy at Peking University International Hospital. This cohort comprised 242 males and 205 females, resulting in a male-to-female ratio of 1.18:1. The ages of the participants ranged from 80 to 95 years, with an average age of 82.76 ± 2.75 years; males constituted approximately 54.1% of the sample. Among these patients, 215 underwent standard colonoscopy, while 232 received painless colonoscopy. Additionally, 185 patients had their procedures conducted during hospitalization, whereas outpatient clinics performed colonoscopies for a total of 262 patients. Notably, colorectal malignancies were diagnosed in 110 individuals, and advanced tumors were identified in198 patients.

In this study, there were 10 colonoscopists, and four of them performed a total of less than 3,000 cases. About 50 cases were performed under the guidance of experienced endoscopists, and no complications such as bleeding and perforation occurred.

The reasons for seeking medical attention among the 447 patients were as follows: history of colon cancer/polyps follow-up (*n* = 97), blood in stool (*n* = 86), change in bowel habits (*n* = 110), abdominal pain/bloating (*n* = 87), weight loss (*n* = 7), positive fecal occult blood (*n* = 28), anemia (*n* = 23), abdominal mass (*n* = 5), and elevated tumor markers (*n* = 4). Demographic data are presented in Table 1.

**Table 1:** Demographic data.

Age	82.0 (80–95)
Female	205/447 (45.9%)
Excellent/good bowel preparation	228 (51.0%)
Cecal intubation rate	364 (81.4%)
Adjusted cecal intubation rate	426 (95.3%)
Total patients with 1 or more polyps	315 (70.5%)

Completed colonoscopy was defined as the successful intubation of the terminal ileum, ileocecal valve, or postoperative ileocolic anastomosis, accompanied by appropriate imaging documentation. It is important to note that some physicians were unable to complete the colonoscopy due to lesions or tumors obstructing the colonic lumen during examination. Consequently, the adjusted completion rate was established as the proportion of obstructive lesions or target lesions identified during all colonoscopies that permitted the colonoscope to reach either the cecum or terminal ileum.

A total of 426 patients completed colonoscopy. Among them, 364 patients had colonoscopy reaching the terminal ileum, ileocecal region and anastomosis. A total of 426 cases of complete colonoscopy were performed, and 21 cases were not completed. Among them, 19 cases were due to poor intestinal preparation and the scope could not be inserted further, and two cases were terminated due to difficulty in inserting the scope. If the cases of inadequate intestinal preparation and stenosis (62 cases) were excluded, the adjusted cecal intubation rate was 95.3%. Due to the more comorbidities and poor physical condition of elderly patients, the technical requirements of enteroscopy are higher.

A total of 37 cases were classified as normal, while the following conditions were observed: 110 cases of colorectal cancer, 315 cases of colorectal polyps, 11 cases of ischemic enteritis, one case of ulcerative colitis, and 10 cases of colon telangiectasia. Additionally, there were 37 postoperative reexaminations for colon cancer patients, along with reports of metastasis in various forms: one case involving lung cancer and intestinal metastasis, another case related to gynecological malignant tumor metastasis, and a single instance of diffuse large B-cell lymphoma. Furthermore, there were also findings indicating colon melanosis in 64 cases and diverticula in 49 cases. Among the cohort diagnosed with colorectal cancer (*n* = 110), the distribution was as follows: rectal cancer accounted for 48 cases; sigmoid colon cancer comprised 28 cases; descending colon cancer was identified in six instances; transverse colon cancer appeared in eight occurrences; and ascending colon cancer was noted in a total of 20 cases. The most prevalent symptom observed in patients with colorectal cancer was the presence of blood in the stool. Among the 85 patients exhibiting this symptom, there were 37 cases diagnosed as colorectal cancer, seven cases of ischemic colitis, one case involving colon polyps with bleeding, one case of ulcerative colitis, two cases of infectious colitis, and three instances of bleeding due to internal hemorrhoids. Additionally, there were 34 cases where no clear bleeding lesions could be identified.

Among the 447 patients evaluated, one individual experienced perforation during polyp removal. Following closure of the wound with a metal clip, this patient exhibited no further symptoms such as fever, abdominal pain, or other discomforts. The patient was subsequently discharged after receiving conservative medical treatment that included antibiotics and intravenous fluid infusion.

Two cases experienced blood in the stool following polyp removal, which improved with conservative medical treatment. Among 232 elderly patients who underwent painless endoscopic examination, one developed aspiration pneumonia. Following anti-infective treatment, the patient’s condition improved, and they were subsequently discharged from the hospital. The overall complication rate associated with colonoscopy and its interventions was 0.67% (Table 2).

**Table 2:** Adverse event.

Adverse event type	Number of cases	Management	Outcome
Perforation	1	Wound closure with a metal clip; Conservative medical treatment (antibiotics, intravenous fluids)	Discharged after symptoms alleviated
Blood in stool	2	Conservative medical treatment	Discharged after symptoms alleviated
Aspiration pneumonia	1	Anti-infective treatment	Discharged after symptoms alleviated
Overall complication rate	0.67% (4/447)		

Univariate analysis concerning colorectal malignant tumors indicated that patients diagnosed with these tumors were older (*P* = 0.000) and had a higher prevalence of alcohol consumption history (*P* = 0.014). However, factors such as gender, smoking status, hyperlipidemia, type 2 diabetes mellitus, body mass index (BMI), and aspirin use did not show significant correlations. In the assessment of risk factors related to advanced tumors, it was found that patients with advanced-stage tumors were also older (*P* = 0.000). No significant differences were observed between groups regarding gender, drinking habits, smoking status, hyperlipidemia, type 2 diabetes mellitus, BMI, or aspirin usage (*P* > 0.005). The relevant data are shown in Tables 3 and 4.

**Table 3:** Demographic characteristics of colonoscopy results: nonmalignant *vs* malignant.

Factor	Total (*n* = 447)	Non-malignant tumors (*n* = 337)	Colorectal malignancies (*n* = 110)	*P*
Age mean ± SD (y)	82.76 ± 2.75	82.35 ± 2.43	83.97 ± 3.27	0.000*
Gender				0.903*
Female	205	154	51	
Male	242	183	59	
Use tobacco				0.168^†^
Never	276	200	76	
Former	141	114	27	
Yes	30	23	7	
Alcohol use				0.014^†^
Never	299	214	85	
Former	100	86	14	
Yes	48	37	11	
Hyperlipidemia^§^				0.074*
Yes	224	177	47	
No	223	160	63	
Low-density lipoprotein^§^	3.26 ± 17.86	2.32 ± 0.84	5.93 ± 35.04	0.309*
Triglycerides^§^	1.31 ± 0.84	1.35 ± 0.89	1.22 ± 0.69	0.181*
cholesterol^§^	4.94 ± 17.25	5.28 ± 20.05	3.98 ± 1.02	0.518*
Glycated hemoglobin^§^	6.83 ± 4.29	7.01 ± 5.09	6.42 ± 1.14	0.448*
Coronary heart disease				0.327*
Yes	122	88	34	
No	325	249	76	
Diabetes				0.136*
Yes	109	88	21	
No	338	249	89	
Hypertension				0.404*
Yes	299	229	70	
No	148	108	40	
BMI§	23.50 ± 3.44	23.60 ± 3.28	23.18 ± 3.89	0.341*
Aspirin				0.379^†^
Never used	231	168	63	
Used intermittently	110	85	25	
Currently taking	106	84	22	
Anaesthetization				0.052*
Yes	232	183	48	
No	216	154	62	

**Notes:**

Statistics are presented as Mean ± SD, Median [P25, P75], Median (min, max), or N (column %).

* *P* values: ANOVA.

† Pearson’s $\chi^2$ test. BMI, body mass index;

§ Data not available for all subjects. Missing values: Hyperlipidemia = 223, BMI = 51, Low-density lipoprotein = 64, Glycated hemoglobin = 300, Triglycerides = 64, Cholesterol = 64.

**Table 4:** Demographic characteristics of colonoscopy findings: non-advanced *vs* advanced neoplasms.

Factor	Total (*n* = 447)	Non-advanced neoplasms (*n* = 249)	Advanced neoplasms (*n* = 198)	*P*
Age	82.76 ± 2.75	82.32 ± 2.39	83.29 ± 3.06	0.000*
Gender				0.194*
Female	205	121	84	
Male	242	128	114	
Use tobacco				0.051^†^
Never	275	149	126	
Former	63	26	37	
Yes	30	11	19	
Alcohol use				0.778^†^
Never	299	153	146	
Former	21	11	10	
Yes	48	22	26	
Hyperlipidemia^§^				0.293*
Yes	224	127	97	
No	223	122	101	
Low-density lipoprotein^§^	3.25 ± 17.86	2.28 ± 0.81	4.36 ± 26.07	0.259*
Triglycerides^§^	1.31 ± 0.84	1.35 ± 0.90	1.27 ± 0.77	0.401*
Cholesterol^§^	4.94 ± 17.25	5.72 ± 23.66	4.05 ± 1.04	0.342*
Diabetes				0.042*
Yes	109	70	39	
No	338	179	159	
Hypertension				0.14*
Yes	299	174	125	
No	148	75	73	
Coronary heart disease				0.779*
Yes	122	69	53	
No	325	180	145	
Glycated hemoglobin^§^	6.83 ± 4.29	6.52 ± 0.93	7.12 ± 5.90	0.401*
BMI^§^	23.50 ± 3.44	23.35 ± 3.23	23.67 ± 3.68	0.356*
Aspirin				0.502^†^
Never used	231	120	111	
Used intermittently	67	40	27	
Currently taking	106	59	47	
Anaesthetization				0.534*
Yes	232	133	99	
No	215	116	99	

**Notes:**

Statistics are presented as Mean ± SD, Median [P25, P75], Median (min, max), or N (column %).

* *P* values: ANOVA.

† Pearson’s $\chi^2$ test. BMI, body mass index;

§ Data not available for all subjects. Missing values: Hyperlipidemia = 223, BMI = 51, Low-density lipoprotein = 64, Glycated hemoglobin = 300, Triglycerides = 64, Cholesterol = 64.

## Discussion

This study encompassed a total of 447 elderly patients aged 80 years and older who were admitted to Peking University International Hospital between November 2015 and June 2024. Among these, there were 110 patients diagnosed with colorectal malignancy. Our findings indicate that age serves as a significant risk factor for both colorectal malignancy and advanced colorectal tumors (*P* = 0.000).

With the continuous extension of global life expectancy, advanced age has emerged as a significant risk factor for the development of colorectal cancer. Colorectal cancer (CRC) currently ranks as the third most prevalent malignant tumor in the world (Morgan et al., 2023). Epidemiological evidence demonstrates that the incidence of colorectal cancer rises exponentially with advancing age (O’Donnell, Hubbard & Jin, 2024). As life expectancy continues to rise among the world population, advanced age emerges as a significant risk factor for colorectal cancer development. Notably, individuals over 65 years old face more than a tenfold increase in CRC risk compared to their younger counterparts (Siegel et al., 2017).

However, an increasing number of patients aged 80 and above are seeking medical attention in clinical settings. With advancing age comes a higher prevalence of underlying comorbidities and declining organ function; consequently, the risks associated with colonoscopy also escalate—particularly concerning intestinal perforation—as well as potential electrolyte imbalances and cardiovascular or cerebrovascular complications related to the procedure (Davidson et al., 2021; Day et al., 2011).

It remains essential for elderly patients aged 80 years and older to undergo endoscopic examinations in clinical practice (Sapci et al., 2022), as some of these patients still possess a considerable life expectancy. All participants in this study underwent endoscopic procedures safely, with no significant increase in the risk of complications compared to other studies (Lu et al., 2025; Hsu et al., 2022; El Halabi et al., 2023); Lu et al.’s (2025) study shows that for screening colonoscopy, the perforation and bleeding rates in patients over 80 were 23.8 and 29.3 cases per 10,000 procedures. Notably, there were no fatalities within 30 days following colonoscopy. The proportions of patients over 80 years old who attended outpatient clinics and those hospitalized for colonoscopy were comparable, indicating that age alone does not preclude patients from receiving this procedure.

Among the elderly population included in this study, the incidence rates of polyps and malignant tumors were found to be 315/447 (70.5%) and 110/447 (24.4%), respectively. The occurrence of tumors and polyps among patients aged 80 years and above was significantly higher than that observed in the general population (Lieberman et al., 2008). Among the 110 patients diagnosed with colorectal malignancies, 69 presented with intestinal stenosis. If timely intervention is not provided, such conditions can lead to severe complications like intestinal obstruction and may hasten the progression of disease. In addition to facilitating accurate diagnosis, colonoscopy also serves as a critical foundation for subsequent surgical interventions and anti-tumor treatments for these patients. The generalizability of these data for the average individual aged 80 years or older is limited due to the inclusion of symptomatic persons in this study who were either 80 years of age or older, or had a previous history of colonic polyps or colon cancer.

The most prevalent symptom observed in the patients participating in this study was a change in bowel habits, followed by abdominal pain or bloating, and the presence of blood in the stool. Weight loss constituted a relatively small proportion of reported symptoms. Notably, similar studies involving individuals aged 80 and above identified weight loss as the most common symptom. Other significant symptoms included alterations in stool thickness, abdominal pain, and hematochezia (Kahi et al., 2014; Esteva et al., 2014).

Our findings indicate that the safety of colonoscopy in elderly patients is acceptable, with no reported deaths within 30 days post-procedure. However, older adults encounter significant challenges related to bowel preparation and examination techniques. This is primarily due to a higher prevalence of underlying comorbidities and the necessity for multiple medications among this population. Additionally, anatomical factors such as longer intestinal lengths and decreased motility further complicate bowel preparation (Cha et al., 2016), thereby impacting the overall efficiency of the examination.

This study suggests that individuals aged 80 years and above often cannot complete a full colon examination predominantly due to intestinal stenosis. Specifically, only 19 cases were unable to proceed with scope insertion owing to inadequate bowel preparation, while two cases had their examinations terminated because of difficulties encountered during scope insertion.

Furthermore, our research indicates that risk factors associated with advanced tumors in individuals over 80 years old are primarily age-related; these factors do not exhibit a strong correlation with gender, smoking status, or alcohol consumption. In contrast, when considering malignant tumors specifically, relevant risk factors include both alcohol consumption and age; however, they show no significant association with gender or conditions such as dyslipidemia, type 2 diabetes mellitus, or hypertension.

It is important to note that some participants originated from outpatient clinics; thus our data collection was retrospective and dependent on the completeness of electronic medical records. Consequently, certain information regarding outpatient smoking habits, alcohol intake levels, blood lipid profiles, and aspirin usage was missing—potentially introducing bias into our final results. Therefore, caution should be exercised when interpreting the outcomes of this study.

Our study indicates that the main risk factor associated with advanced tumors in people over 80 years old is age, and has little correlation with gender, smoking, and drinking. However, in malignant tumors, the relevant risk factors are drinking and age, and have little correlation with gender, blood lipid levels, type 2 diabetes mellitus, and hypertension. Among patients with colorectal malignancies, people who never drink have a higher incidence rate. Alcohol consumption is a protective factor for colorectal malignancies, which is inconsistent with previous research results (Veettil et al., 2021). Considering that the inconsistency between our research results and previous literature may be due to the small number of people enrolled in the study, the lack of drinking information for some patients due to the incomplete recording of the medical records of some patients, and our data do not have analysis on the amount of drinking and the number of years of drinking, it needs to be interpreted with caution. Our study did not find any correlation between factors such as hypertension, type 2 diabetes mellitus, coronary heart disease, hyperlipidemia, gender and colorectal malignancies. Age is the only risk factor, which is consistent with the conclusions of many previous papers. Therefore, colonoscopy is very necessary for elderly people aged 80 years and above who have clinical symptoms (Veettil et al., 2021; Nee, Chippendale & Feuerstein, 2020). In this study, the utilization of sedation for colonoscopy among elderly patients was found to be lower 232/447 (51.9%) compared to the corresponding rate observed in the same endoscopy center during the same period. This reduction can be attributed to factors such as advanced age and underlying comorbidities, which raise concerns among anesthesiologists regarding anesthesia-related complications and their reluctance to administer anesthesia treatment to these patients. Consequently, certain individuals experience heightened anxiety towards undergoing colonoscopy. After rigorous evaluation, some of these patients can safely complete colonoscopy, even painless colonoscopy.

However, there remains considerable controversy regarding whether asymptomatic elderly individuals aged 80 years and older should undergo colonoscopy screening. The guidelines recently issued by the US Preventive Services Task Force recommend against CRC screening for individuals over 85 years of age, highlighting that the benefits of screening for patients between 76 and 85 years old are uncertain (U.S. Preventive Services Task Force, 2008). Further research is needed to determine whether colonoscopy screening is beneficial for this asymptomatic population.

For patients aged 80 years or older, literature indicates that the risk of all-cause complications has increased to 34.8 per 1,000 colonoscopies (U.S. Preventive Services Task Force, 2008; Cheong, Faye & Shaukat, 2023). Compared to patients aged 65 years or older, those aged 80 and above exhibit a higher risk of perforation (Day et al., 2011). In our study, the overall complication rate related to colonoscopy was found to be 0.67%, which is lower than reported in similar studies; notably, no deaths occurred within 30 days following the procedure.

Our study does have certain limitations due to its retrospective design and reliance on the accuracy and completeness of electronic medical records. Some participants were sourced from outpatient clinics where data concerning smoking habits, alcohol consumption, blood lipid levels, and aspirin use were incomplete; such omissions may introduce bias into our final results. Furthermore, as most current guidelines do not advocate for colonoscopy screening in individuals over the age of 75 years (Wolf et al., 2018), our study exclusively included symptomatic patients; thus, findings may not be fully generalizable to asymptomatic individuals over the age of 80.

This study did not include data on endoscopic quality indicators or the number of colonoscopies performed by individual endoscopists, which may have influenced the final outcomes. Although more than half of the participating endoscopists had extensive experience, individual polyp and adenoma detection rates were not documented. Previous evidence indicates that endoscopist experience should be reflected in these detection rates. However, experienced endoscopists often perform a higher volume of procedures, and increased workload may lead to procedural fatigue, potentially reducing lesion detection rates.

Furthermore, the high cecal intubation rate (95.3%) observed in this study may be attributed to the expertise of the endoscopists. Nevertheless, variations in lesion detection rates among different physicians were not analyzed, which could introduce outcome bias.

Only 51.9% (232/447) of patients received sedation during the procedure, significantly lower than the typical rate (>90%). The absence of sedation may compromise patient tolerance and affect procedural completion. In this study, most malignant tumors were located in the distal colon (48 cases in the rectum and 28 in the sigmoid colon), with only 34 cases (30.9%) found in the proximal colon. This contrasts with previous findings suggesting a higher prevalence of proximal tumors in elderly populations. These discrepancies may stem from differences in ethnicity or inclusion criteria. For certain high-risk elderly individuals, our findings support the prioritization of sigmoidoscopy to minimize procedural risks.

Among the 447 patients aged ≥80 years, the severe complication rate was low at 0.67% (1 perforation and 2 bleeding events), with no deaths within 30 days. The adjusted procedural completion rate was 95.3%, exceeding the 70–89% reported in the literature. To our knowledge, this is the first study demonstrating that colonoscopy can be as safe in elderly patients as in younger individuals when performed by experienced operators, challenging the traditional belief that advanced age is a contraindication for the procedure.

An additional novel finding was that a history of alcohol consumption was associated with a lower risk of malignant lesions (*P* = 0.014), contradicting the conventional view that alcohol is a CRC risk factor. This observation may reflect survivor bias—where long-term drinkers are less likely to reach advanced age—or a potential protective effect of low-dose alcohol exposure. Further prospective studies are needed to validate this association.

Moreover, both the polyp detection rate (70.5%) and the malignant tumor detection rate (24.6%) in this cohort far exceeded those in the general population (polyps: 20–30%; malignancies: 0.5–1%). These results confirm the high intestinal tumor burden in symptomatic individuals aged ≥80 years and highlight the effectiveness of colonoscopy in identifying clinically significant lesions requiring intervention.

Additionally, this investigation did not encompass information pertaining to endoscopic quality indicators or the number of procedures performed by individual endoscopists—factors that could influence outcomes but remain unexplained in our analysis. Therefore, caution should be exercised when interpreting these results.

Nevertheless, it is crucial to acknowledge two further limitations. Firstly, the data collection for this research was finalized in June 2024. This implies that the findings are grounded in information that is currently over a year old. Although this temporal gap does not nullify the core findings, it accentuates the necessity for more up-to-date data to corroborate these observations within a rapidly changing clinical landscape. Secondly, the relatively modest sample size (*n* = 447) might circumscribe the statistical power of the analyses and the generalizability of the results to the broader cohort of individuals aged 80 and older. These aspects ought to be taken into account when interpreting the findings.

## Conclusion

This study confirms that advanced age (≥80 years) is an independent risk factor for colorectal malignancies (*P* = 0.000) and advanced neoplasms. Among 447 symptomatic elderly patients, the detection rates of colorectal polyps (70.5%) and malignant tumors (24.6%) were significantly higher than those observed in the general population. When performed by experienced operators, the adjusted examination completion rate reached 95.3%, with a total complication rate of 0.67% and no mortality within 30 days post-procedure. A notable but controversial finding was that a history of alcohol consumption may be associated with a reduced risk of malignancy (*P* = 0.014); however, this observation requires further validation through prospective studies to assess potential dose-response relationships and the influence of survivor bias. These findings support the timely implementation of colonoscopy for symptomatic elderly patients, particularly those at high risk for distal tumors. It is also recommended to optimize procedural strategies, such as prioritizing sigmoidoscopy for high-risk individuals. The limitations of this study stem from its retrospective design, which resulted in incomplete data on certain risk factors. Therefore, the conclusions are primarily applicable to symptomatic individuals, and the potential benefits of screening in asymptomatic older adults warrant further investigation.

## Supplemental Information

10.7717/peerj.20404/supp-1 Supplemental Information 1 STROBE Statement.

10.7717/peerj.20404/supp-2 Supplemental Information 2 Data on colonoscopy for patients over 80 years old. Ages, genders, comorbidities, colonoscopy reports and pathological information, *etc*.

## Additional Information and Declarations

### Data Availability

The following information was supplied regarding data availability:

The original data is available in the Supplemental File.
