# Supplementary material for: Outcomes and safety of colonoscopy in elderly patients aged 80 years and older: a retrospective cohort study
Source: PeerJ. 2025 Nov 19;13:e20404. doi: 10.7717/peerj.20404 (PMC12640131; doi:10.7717/peerj.20404)
Supplement: Supplemental Information 1 [file peerj-13-20404-s001.docx]

STROBE Statement - Strengthening the Reporting of Observational Studies in Epidemiology

1. Title and Abstract

1.1 Title

“Analyzing the Outcomes and Safety of Colonoscopy in Elderly Patients Aged 80 Years and Older” clearly indicates the study population (elderly patients aged 80 and older), the exposure (colonoscopy), and the outcomes (outcomes and safety).

1.2 Abstract

- **Background**: Briefly presents the background that colorectal tumor incidence increases with age and colonoscopy is crucial for early detection, but there are challenges in performing it on octogenarians due to their complex health conditions.

- **Methods**: Mentions it is a retrospective study including 447 elderly patients who underwent colorectal examinations from 2016 - 2024 at Peking University International Hospital. Data on various factors were collected, and risk factors for different types of colorectal tumors were compared.

- **Results**: Reports the successful procedure rate, the distribution of diagnoses (e.g., normal, polyps, malignant tumors), the complication rate, and significant risk factors (age for colorectal malignancies and advanced tumors; alcohol consumption history for colorectal malignancies).

- **Conclusions**: Concludes that colonoscopy is effective and safe for this population, age is a significant risk factor, but the study has limitations. The abstract provides a concise summary of the key aspects of the study.

2. Introduction

2.1 Background and Rationale

- Describes the global aging population issue and the increasing incidence of colorectal tumors with age. Colonoscopy is introduced as the gold standard for diagnosing colorectal cancer, but it has higher risks in elderly patients. This sets the stage for the study's aim to evaluate colonoscopy in 80 - year - olds and older.

- Cites relevant literature to support the background information, such as the ranking of colorectal tumors as the third most common malignant neoplasms globally and the decline in CRC incidence and mortality in the US due to enhanced screening.

2.2 Objectives

States the clear objective of evaluating the impact of colonoscopy and analyzing its results in adults aged 80 years and older.

3. Methods

3.1 Study Design

- Clearly states that it is a retrospective study. This design is appropriate considering the nature of the research question, as it allows for the collection of data from past medical records of patients who have already undergone colonoscopy.

3.2 Setting

- Specifies the study was conducted at the Endoscopy Center of Peking University International Hospital from 2016 to June 2024. The setting is well - defined, which helps in understanding the context of the study and the patient population.

3.3 Participants

- **Inclusion Criteria**: Involved patients aged 80 years and older who underwent colonoscopy at the specified center during the study period. Only the initial colonoscopy data of each patient were included.

- **Exclusion Criteria**: Patients with clear intestinal perforation, significant cardiopulmonary dysfunction, or mental disorders that impaired cooperation during the colonoscopy were excluded. These criteria are relevant and help in defining a homogeneous study population.

3.4 Variables

- Lists a comprehensive set of variables including age, gender, smoking and drinking history, family history of colorectal cancer, various medical conditions (hyperlipidemia, diabetes mellitus, etc.), colonoscopy - related factors (indications, complications, etc.), and mortality within 30 days post - procedure. The definitions for colorectal malignancy and advanced neoplasms are also provided.

3.5 Data Sources/Measurement

- Data were collected from the electronic medical records of selected patients, including multiple types of records such as clinical notes, examination reports, etc. The collection method is practical for a retrospective study, but the potential limitation of relying on the completeness of these records is acknowledged.

3.6 Bias

- Recognizes potential biases due to the retrospective nature of the study and the incomplete data from outpatient clinics regarding smoking habits, alcohol intake, etc. This shows awareness of the limitations that could affect the validity of the results.

3.7 Study Size

- The sample size of 447 patients is clearly stated. While not justified in terms of power analysis (common in retrospective studies), the size is presented for transparency.

3.8 Quantitative Variables

- For normally distributed variables, data are presented as mean plus standard deviation; for non - normally distributed variables, as the median. Categorical variables are expressed as absolute numbers with percentages. The statistical methods used for comparing these variables (χ² test, Fisher's exact test, t - test, Mann - Whitney U test) are appropriate and clearly described.

3.9 Statistical Methods

- All calculations were performed using SPSS version 26.0 software. The use of a well - known statistical software and the description of the specific statistical tests for different types of variables enhance the reproducibility of the study.

4. Results

4.1 Participant Flow

- Reports the total number of patients (447), the number of males and females, and the age range. Details the number of patients who underwent standard and painless colonoscopy, as well as those who had the procedure during hospitalization or as outpatients.

- Presents the reasons for seeking medical attention, the number of completed and uncompleted colonoscopies, and the reasons for non - completion. The adjusted cecal intubation rate is also provided.

4.2 Baseline Data

- Demographic data such as age, gender distribution, and medical history are presented in tables, which allows for easy comparison. These data are relevant for understanding the characteristics of the study population.

4.3 Numbers Analyzed

- Clearly states the number of patients included in different analyses, for example, the number of patients with different diagnoses (colorectal cancer, polyps, etc.).

4.4 Outcome Measures

- Reports the incidence of different conditions (colorectal cancer, polyps, etc.), adverse events (perforation, bleeding), and the overall complication rate. The results are presented in a clear and organized manner.

4.5 Main Results

- Univariate analysis results for risk factors of colorectal malignant tumors and advanced tumors are presented. The significant factors (age, alcohol consumption history) are clearly stated, along with the non - significant factors.

4.6 Other Analyses

- No other complex subgroup or sensitivity analyses are reported, which is acceptable given the study design, but it may limit the depth of understanding of the results.

5. Discussion

5.1 Key Results

- Summarizes the key findings that age is a significant risk factor for colorectal malignancy and advanced tumors. Discusses the high incidence of polyps and malignant tumors in this population and the acceptable safety of colonoscopy.

- Compares the results with previous literature, highlighting both similarities and differences, such as the inconsistent finding regarding alcohol consumption as a risk factor.

5.2 Limitations

- Acknowledges multiple limitations including the retrospective design, reliance on electronic medical records for data collection, potential bias from incomplete data, and the inability to generalize the results to asymptomatic individuals over 80.

- Also mentions the lack of information on endoscopic quality indicators and the number of procedures per endoscopist, which could have influenced the outcomes.

5.3 Interpretation

- Interprets the results in the context of the limitations. Suggests that colonoscopy is still valuable for elderly patients with clinical symptoms, but further research is needed for asymptomatic elderly individuals.

- Discusses the controversy regarding colonoscopy screening in asymptomatic octogenarians based on current guidelines.

5.4 Generality

- Recognizes the limited generalizability of the study due to the inclusion of only symptomatic patients and the potential bias in data collection.

6. Other Information

6.1 Funding

- No information on funding is provided. If there was no external funding, it should be stated for transparency. If there was funding, details about the source and any potential conflicts of interest should be disclosed.

6.2 Ethical Approval

- The study adhered to the ethical guidelines of the 1975 Declaration of Helsinki and was approved by the Biomedical Ethics Committee of Peking University International Hospital on October 15, 2024, with the approval number 2024 - KY - 0093 - 01. The waiver of informed consent due to the retrospective nature of the study and lack of obvious potential harm is also explained.
